# Electrospun PVP Fibers as Carriers of Ca^2+^ Ions to Improve the Osteoinductivity of Titanium-Based Dental Implants

**DOI:** 10.3390/molecules29174181

**Published:** 2024-09-03

**Authors:** Janina Roknić, Ines Despotović, Jozefina Katić, Željka Petrović

**Affiliations:** 1 Department of Electrochemistry, Faculty of Chemical Engineering and Technology, University of Zagreb, Marulićev trg 19, 10000 Zagreb, Croatia; roknic57@gmail.com (J.R.); jkatic@fkit.unizg.hr (J.K.); 2 Division of Physical Chemistry, Ruđer Bošković Institute, Bijenička Cesta 54, 10002 Zagreb, Croatia; 3 Division of Materials Chemistry, Ruđer Bošković Institute, Bijenička Cesta 54, 10002 Zagreb, Croatia

**Keywords:** titanium, electrospinning, polyvinylpyrrolidone, Ca^2+^ ions, EIS, DFT

## Abstract

Although titanium and its alloys are widely used as dental implants, they cannot induce the formation of new bone around the implant, which is a basis for the functional integrity and long-term stability of implants. This study focused on the functionalization of the titanium/titanium oxide surface as the gold standard for dental implants, with electrospun composite fibers consisting of polyvinylpyrrolidone and Ca^2+^ ions. Polymer fibers as carriers of Ca^2+^ ions should gradually dissolve, releasing Ca^2+^ ions into the environment of the implant when it is immersed in a model electrolyte of artificial saliva. Scanning electron microscopy, energy dispersive X-ray spectroscopy and attenuated total reflectance Fourier transform infrared spectroscopy confirmed the successful formation of a porous network of composite fibers on the titanium/titanium oxide surface. The mechanism of the formation of the composite fibers was investigated in detail by quantum chemical calculations at the density functional theory level based on the simulation of possible molecular interactions between Ca^2+^ ions, polymer fibers and titanium substrate. During the 7-day immersion of the functionalized titanium in artificial saliva, the processes on the titanium/titanium oxide/composite fibers/artificial saliva interface were monitored by electrochemical impedance spectroscopy. It can be concluded from all the results that the composite fibers formed on titanium have application potential for the development of osteoinductive and thus more biocompatible dental implants.

## 1. Introduction

As we live in an age in which quality of life plays an important role, the demand for various types of implants is rising significantly [1,2]. With the increasing number of implants, the incidence of adverse reactions of the body to the inserted implant, such as inflammation, allergic reactions or failed osseointegration, inevitably rises [3,4,5]. For this reason, numerous possibilities have been researched to better adapt the surface of the implant to the human body [6].

One of the possibilities are novel coatings with osteoinductive and/or antimicrobial properties, which can be divided into two groups: inorganic and organic coatings [6]. Hydroxyapatite (HAp) and calcium phosphate (CaP) are the most commonly used inorganic coatings, whose main function is to improve the osseointegration of implants [7]. Organic coatings such as drug-releasing [8,9] or anti-fouling coatings [10] are important due to their antibacterial effect or their ability to prevent the build-up of proteins and microorganisms and inhibit the adhesion of bacteria. A porous N-halamine polymer coating has been found to have a long-lasting, renewable antibacterial efficacy for the Ti surface [11]. Recently, composite coatings of different polymers and active molecules have become increasingly popular as a system for transporting the drug to a target site (drug delivery), with the polymer acting as a carrier of the active molecule [12,13,14]. It has been reported that tetracycline (TCH), as an active substance incorporated into various polymer fibers [poly (dl-lactide, PLA; polycaprolactone, PCL); type B gelatin (GEL)], plays a role as an antimicrobial surface modifier and osteogenic inducer for Ti implants [15,16]. Cellulose acetate fibers with daptomycin have been applied to stainless steel as a coating for drug delivery [17]. The polymeric fibers of chitosan (CS) and polyethylene oxide (PEO) loaded with vancomycin have successfully reduced the concentration of bacteria near the implant surface [18].

One of the most suitable and promising polymers as a carrier of active compounds is the polymer polyvinylpyrrolidone (PVP) due to its versatility and special properties [14,19]. It is a hydrophilic polymer, it has very good solubility in various solvents, and is temperature-resistant, pH-stable, available in various molecular weights, biocompatible and non-toxic [20,21]. PVP can be produced in the form of hydrogels [22], oral tablets [23], films [24] or electrospun fibers [25,26]. It is widely used in cosmetics, food, adhesives, textiles and, more recently, as a biomaterial such as a binding agent in pharmaceutical tablets, hydrogels for wound dressings, disinfectants and blood plasma substitutes [27]. The disadvantage of using PVP as a biomaterial is that it is not biodegraded in the body, and the problem of its residues should be solved appropriately [14]. The PVP in the form of electrospun fibers loaded with ibuprofen were synthesized as an oral, fast-dissolving drug system [25,26]. The addition of zinc oxide and silver to the nanofibers of the PVP/PCL polymer system has shown improved antibacterial activity [28]. If the appropriate fiber properties are achieved, PVP can also be mixed with another polymer [29].

Composite coatings in the form of long fibers are mostly used for the above-mentioned drug delivery. Although fiber coatings can be produced using various techniques such as template synthesis [30] or the seeding of nanofibers [31], the electrospinning technique stands out due to its numerous advantages [32]. The electrospinning method can be used to produce continuous organic, inorganic or composite micro/nanofibers with a large surface area and the potential for subsequent functionalization. The process is also relatively inexpensive. However, the production of fibers with the desired morphology and properties requires meticulously taking into account different synthesis parameters (applied voltage, flow rate, distance between metal needle and collector), the concentration and the viscosity of the solution, and the humidity and ambient temperature [33,34]. The electrospun fibers are already widely used in biomedicine for the treatment of wounds, the immobilization of enzymes, and in dentistry for the regeneration of teeth and the prevention of dental caries [32,35,36,37].

It is known that a dental implant can only remain in the jaw for a long time if it forms a stable bond with the surrounding bone. The aim of this study was to stimulate the formation of new bone, i.e., the osseointegration of dental implants. Therefore, the surface of the titanium (Ti) was modified with a biodegradable coating rich in Ca^2+^ ions. It is expected that the coating will dissolve after implantation and release Ca^2+^ ions to the implant environment, thus contributing to successful osseointegration. With this in mind, the PVP polymer was selected as a material that is highly soluble in various media as a carrier for active Ca^2+^ ions. Ca^2+^ ions were chosen because they play an important role in controlling the proliferation and differentiation of osteoblasts and the bone formation process, as numerous in vitro studies have shown [38,39,40,41]. The osseointegration and bone formation examined in vivo were also improved when the Ti6Al4V implant surface was enriched with calcium ions [42]. For this purpose, the electrospinning method was used to synthesize a biodegradable coating in the form of composite fibers (PVP+Ca^2+^) on an oxide-coated titanium surface.

Although PVP fibers in combination with Ca compounds such as calcium phosphates or hydroxyapatite have been studied for dental applications [43,44], the combination with “free” Ca^2+^ ions for dental implants is not yet found in the literature. Therefore, the synthesis of the biodegradable (PVP+Ca^2+^) fibers is a novelty and contributes to the development of osteoinductive implants. An additional contribution of the work is a detailed investigation of the mechanism of the formation of composite (PVP+Ca^2+^) fibers on the titanium surface using density functional theory (DFT). For the modeling of the solid surface, the cluster approach, i.e., a fragment of a lattice of a finite size, was utilized, which may be the cause of the possible limitations of the model itself [45]. However, employed clusters provide credible models for the investigation of composite fibers–surface interactions due to the localized effects of surface modifier binding. The dissolution of the composite fibers in interaction with artificial saliva was monitored by electrochemical impedance spectroscopy (EIS) over 7 days, which represents the initial phase of the osseointegration of the implant.

## 2. Results and Discussion

### 2.1. Chemical and Morphological Characterization of Titanium Samples

**Figure 1:**
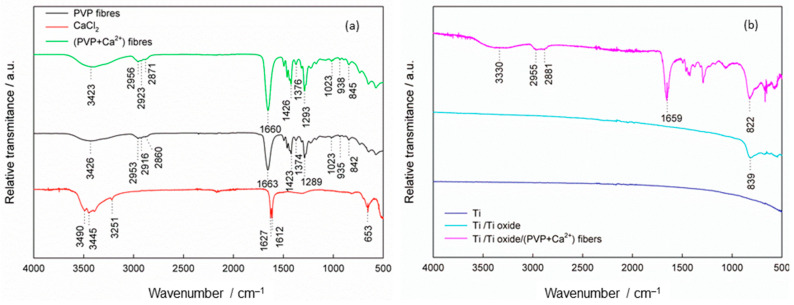
The ATR-FTIR spectra of (**a**) CaCl_2_ salt, PVP and (PVP+Ca^2+^) fibers; (**b**) freshly prepared Ti, Ti modified with thermally prepared oxide film (Ti/Ti oxide) and Ti modified with the composite fibers [Ti/Ti oxide/(PVP+Ca^2+^) fibers].

To confirm the binding of the (PVP+Ca^2+^) fibers to the thermally oxidized Ti, a chemical analysis was carried out using attenuated total reflectance Fourier transform infrared (ATR-FTIR) spectroscopy, Figure 1. For comparison, the spectra of bare PVP and (PVP+Ca^2+^) fibers, CaCl_2_, were recorded, as well as Ti samples with and without oxide film. As can be seen in Figure 1a, the spectra of PVP and (PVP+Ca^2+^) fibers are very similar, so the response for the (PVP+Ca^2+^) fibers is described in more detail. A brief observation indicates the dominance of the organic phase in the structure of both fibers, as a large number of bands are present. A broad band in the wavenumber range from 3600 to 2500 cm^−1^ and with a peak at 3416 cm^−1^ was attributed to stretching vibrations of OH groups connected by hydrogen bonds, indicating the presence of adsorbed water. Bands caused by asymmetric and symmetric stretching vibrations of CH_2_ groups in the pyrrole ring appear at 2956 and 2923 cm^−1^, and a band at 1660 cm^−1^ is assigned to stretching vibrations of the carbonyl and C-N groups [46,47,48]. The band at 2871 cm^−1^ is characteristic of C-H stretching. The deformation vibrational bands of the CH_2_ groups are located at 1426 cm^−1^, and the same bands in the case of the CH groups are located at 1376 cm^−1^ [47,48]. The band at 1293 cm^−1^ appears due to deformation vibrations of the C-N bond or due to the wagging of the C-H bonds in the CH_2_ group. The band at 1023 cm^−1^ can be attributed to C-N bond or C-C bond vibrations and the rocking of the CH_2_ group [46,47]. At 938 cm^−1^ there are vibrations in C-C bonds, and at 845 cm^−1^ there are deformation vibrations in the CH_2_ group or stretching in the C-C-H plane [46,47].

The band shift of the carbonyl group of the (PVP+Ca^2+^) fibers compared to the band position of the carbonyl group of the PVP fibers points to the interaction between the carbonyl oxygen of PVP and Ca^2+^ ions. The carbonyl band of PVP is visible at 1663 cm^−1^, while it shifts to 1660 cm^−1^ for the (PVP+Ca^2+^) fibers. A similar observation was reported by H. Liu et al. [49]. An additional shift is visible when (PVP+Ca^2+^) fibers are bound to the Ti/Ti oxide surface (1659 cm^−1^, Figure 1b).

The ATR-FTIR spectrum of the starting salt CaCl_2_, which is the source of the Ca^2+^ ions, shows bands at 3490, 3445, 3251, 1627 and 1612 cm^−1^, which relate to the stretching vibrations of the OH functional group, while the band at 653 cm^−1^ is characteristic of the stretching vibration of the Ca-O bond [50]. The appearance of this band can be attributed to the presence of Ca(OH)_2_, which is present in small quantities (manufacturer’s specification), and to the hygroscopicity of the salt.

Figure 1b shows the spectra of freshly polished Ti, Ti with thermally prepared oxide film (Ti/Ti oxide) and Ti with an oxide film and composite fibers [Ti/Ti oxide/(PVP+Ca^2+^) fibers]. It can be seen that each modification makes the ATR-FTIR response of the Ti more complex compared to the freshly prepared Ti surface. A Ti sample heat-treated at 450 °C shows a distinct band at 839 cm^−1^, which can be attributed to the stretching vibrations of the Ti-O bond that normally occur in the wavenumber range from 1000 to 350 cm^−1^ [51,52]. The appearance of this band points to the formation of an oxide film on the Ti surface. After the modification of the Ti/Ti oxide sample with electrospun fibers [(PVP+Ca^2+^) fibers], the response is even more complex, indicating their successful formation on the Ti/Ti oxide surface. The spectrum is dominated by bands in the range ~1700–1000 cm^−1^ originating from the PVP (see Figure 1a). A distinct band at 822 cm^−1^, which is assigned to the oxide film, is shifted compared to the bare Ti/Ti oxide (839 cm^−1^) due to the interaction between oxide and fibers. The observed ATR-FTIR bands confirm the successful formation of the (PVP+Ca^2+^) fibers on the Ti/Ti oxide surface.

**Figure 2:**
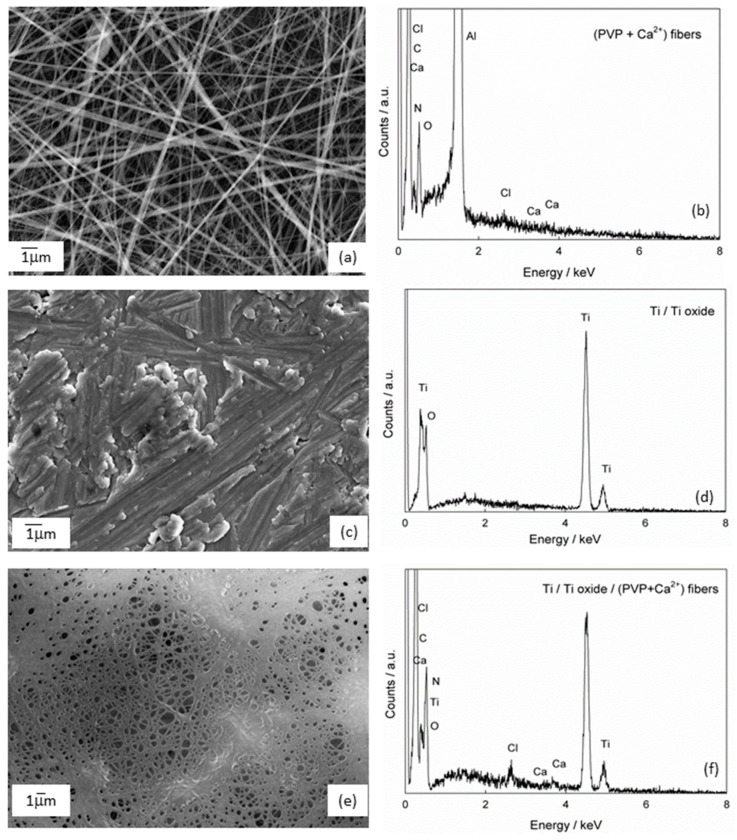
The SEM images and corresponding EDS spectra of (**a**,**b**) (PVP+Ca^2+^) fibers; (**c**,**d**) Ti modified with thermally prepared oxide film (Ti/Ti oxide) and (**e**,**f**) Ti modified with the composite fibers [Ti/Ti oxide/(PVP+Ca^2+^) fibers].

The morphology of the Ti sample without and with the formed composite fibers [Ti/Ti oxide/(PVP+Ca^2+^) fibers] was examined by field emission scanning electron microscopy (FE-SEM); Figure 2. In addition to the FE-SEM, an elemental analysis was also carried out using energy dispersive X-ray spectroscopy (EDS). Before the fibers were formed on the Ti/Ti oxide surface, the starting solution was electrospun on the Al foil to test the formation of the fiber structure. The successfully formed fiber structure under the given experimental conditions (Figure 2a,b; in the EDS spectrum, Al comes from Al foil) was a justification for the following step of forming composite fibers on the Ti/Ti oxide sample. 

The morphology of the bare Ti/Ti oxide sample was investigated, and the result was shown in Figure 2c,d. The SEM image of the Ti/Ti oxide shows an inhomogeneous surface covered with a layer of varying thickness. Grooves can be seen, which are due to the grinding of the sample during pretreatment (see Section 3.1). EDS analysis confirms the presence of titanium and oxygen, indicating the formation of an oxide film on the sample surface as a result of the thermal treatment. After the formation of (PVP+Ca^2+^) fibers on the Ti/Ti oxide surface, a porous network of fibers is visible, Figure 2e. Dense, sticky fibers can be observed in some places. It can be concluded that the 3 min electrospinning has formed an inhomogeneous porous network through whose pores the oxide film is visible. The EDS analysis (Figure 2f) confirms the presence of elements of the oxide film (Ti and O) and the composite fibers (C, N, Ca, Cl).

It can also be concluded from the above results that the electrical conductivity of the substrate has a considerable influence on the morphology of the fibers. The fibers that were electrospun on a highly conductive aluminum substrate are clearly defined and have the shape of long, homogeneous fibers (Figure 2a). In contrast, the fibers produced on a poorly conductive substrate, titanium coated with a thin oxide film, look sticky and lose the shape of the fibers in some places (Figure 2c).

ATR-FTIR, SEM and EDS techniques have shown that under selected experimental conditions, it is possible to form a porous network of (PVP+Ca^2+^) fibers on the Ti/Ti oxide surface. Since these fibers were synthesized under the assumption that they release Ca^2+^ ions to the model electrolyte, the processes at the Ti/Ti oxide/(PVP+Ca^2+^) fibers/artificial saliva interface were investigated using electrochemical impedance spectroscopy (Section 2.3). Furthermore, all molecular interactions between Ca^2+^ ions, PVP molecules and Ti/Ti oxide were investigated in detail using DFT quantum chemical calculations to predict a possible mechanism of fiber formation as well as expected Ca^2+^ ion release potential (see Section 2.2).

### 2.2. The Mechanism of Interaction of Calcium Ions with PVP Fibers as Well as (PVP+Ca^2+^) Fibers with Titanium

In order to gain insight at the molecular level and an understanding of the possible interactions between Ca^2+^ ions and PVP polymer, as well as the interactions between (PVP+Ca^2+^) composite fibers and Ti substrate, a detailed theoretical study with quantum chemical calculations at the density functional theory (DFT) level was performed. When dealing with large chemical systems such as polymer molecules, they can be truncated to their lower oligomeric forms to obtain model systems that allow for a more systematic and detailed structural investigation. Such a shortening leads to a model system that represents a compromise between chemically realistic systems and calculations and enables a drastic reduction in computational time. In the calculations performed, the PVP polymer was therefore modeled by short PVP-tetramer chains, as these appeared long enough to account for all possible interactions with the Ca^2+^ ions, and at the same time small enough to perform the calculations efficiently.

**Figure 3:**
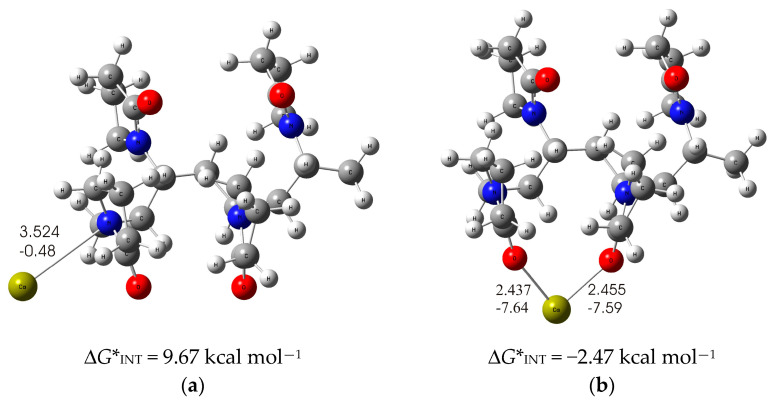
The optimized structures of (**a**) PVP-Ca-I, in which the PVP-Ca bond is established via a nitrogen atom; (**b**) PVP-Ca-II, in which the PVP-Ca bond is established via oxygen atoms of the carbonyl groups. The bond distances are given in Å. The bond energies are given in kcal mol^−1^. O—red, C—gray, N—blue, H—white, Ca—yellow-green.

As is known from the literature, PVP can coordinate metal cations due to the high affinity of pyrrolidone residues to metal cations [53]. The metal–PVP interaction can occur either via the carbonyl oxygen or the nitrogen atom on the five-membered nitrogen-containing heterocycles of PVP [54]. Therefore, an imperative task of this research was to investigate these two possible modes of Ca^2+^ ion coordination with a DFT-optimized structure of the PVP tetramer in the ethanol solvent to find out the most probable mode of binding of Ca^2+^ ions by the PVP polymer. The structures with the lowest energy for each binding mode were calculated and are shown in Figure 3.

In the significantly less stable PVP-Ca-I structure (Figure 3a), the formation of PVP-Ca^2+^ complex is accomplished via the nitrogen atom of the pyrrolidone ring, whereby the free electron pair of the nitrogen atom of the pyrrolidone ring is involved in the bond with the calcium ion and forms a coordinate Ca-N bond (*d*_Ca⋯N_ = 3.524 Å; *E*_Ca⋯N_ = −0.48 kcal mol^−1^). The formed Ca-N bond is attributed to an ionic type of interaction according to ∇^2^*ρ*(*rc*) > 0 and *H*(*rc*) > 0 from the topological analysis of the electron density distribution. The binding of the calcium ion to the two pyrrolidone rings proves to be an endergonic process with a positive Gibbs free interaction energy of ΔG*_INT_ = 9.67 kcal mol^−1^.

If the Ca^2+^ ions bind to the PVP via the carbonyl oxygen atoms of the pyrrolidone ring, this results in a much more stable structure. In this structure, the Ca^2+^ ions bind to two oxygen atoms of the carbonyl groups of the two pyrrolidone rings, as shown in Figure 3b. The two coordinate C-O bonds formed (*d*_Ca···O_ = 2.437 Å; *E*_Ca⋯O_ = −7.59 kcal mol^−1^ and *d*_Ca⋯O_ = 2.455 Å; *E*_Ca⋯O_ = −7.64 kcal mol^−1^) are attributed to an ionic type of interaction according to ∇^2^*ρ*(*rc*) > 0 and *H*(*rc*) > 0. One should note the much stronger Ca-O bonds in the PVP-Ca-II structure compared to the Ca-N bonds in the PVP-Ca-I structure. Accordingly, the calcium binding to the PVP tetramer appears to be more favorable in the PVP Ca-II structure, as it is an exergonic process with releasing a Gibbs free energy of ΔG*_INT_ = −2.47 kcal mol^−1^. Based on these results, we can clearly conclude that the most likely mode of binding is in accordance with the PVP-Ca-II structure, i.e., via the carbonyl oxygen atoms of the pyrrolidone rings.

**Figure 4:**
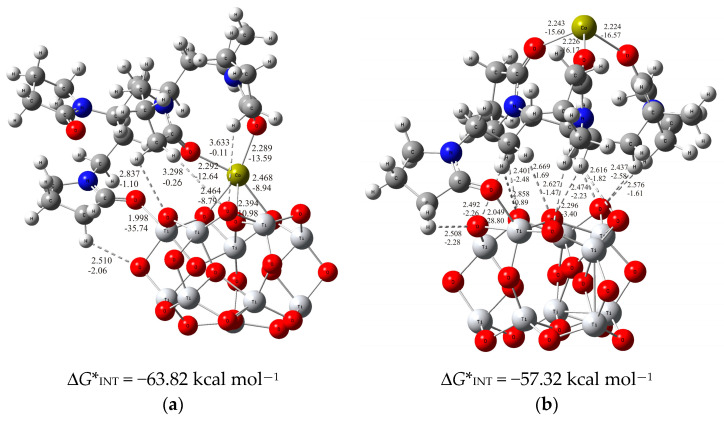
The optimized structures of (**a**) PVP-Ca-TiO_2_-I, in which the Ca^2+^ is complexed in between PVP layer and TiO_2_ surface, (**b**) PVP-Ca-TiO_2_-II, in which the Ca^2+^ is bound on the top of PVP layer. The bond distances are given in Å. The bond energies are given in kcal mol^−1^. O—red, C—gray, N—blue, H—white, Ca—yellow-green.

In order to obtain information about the chemical binding of the PVP-Ca^2+^ species to the titanium oxide surface, a thorough analysis of the different possible binding modes was performed. Following the previous analysis, the PVP-tetramer short-chain-carrying Ca^2+^ ions was used for the PVP-Ca^2+^ species in the simulations performed, and the (TiO_2_)_10_ nanocluster [55] was selected as the theoretical model for the simulation of the metal oxide film on the titanium surface. It must be mentioned at this point that the influence of the solvent was not considered in the simulation of PVP-Ca^2+^ species binding to the TiO_2_ surface due to the given experimental conditions, which cause the immediate evaporation of the solvent. Based on the DFT calculations, the most probable interaction modes were predicted, which are shown in Figure 4.

As shown by the calculations, the formation of the PVP fibers carrying Ca^2+^ ions on the TiO_2_ surface most likely occurs through two energetically competing structures: in one, the Ca^2+^ ions are simultaneously bound to the inorganic TiO_2_ surface and PVP molecule being placed in between (PVP-Ca-TiO_2_-I structure) these two layers, and in the other, the Ca^2+^ ions are bound on the top of PVP layer (PVP-Ca-TiO_2_-II structure). In the PVP-Ca-TiO_2_-I structure, molecular modeling predicts the binding of the PVP-tetramer to the (TiO_2_)_10_ cluster via the pyrrolidone ring of the PVP molecule, which is achieved by the strong coordinate O_carbonyl_-Ti bond (*d*_O⋯Ti_ = 1.998 Å; *E*_O⋯Ti_ = −35.74 kcal mol^−1^) being attributed to an ionic type of interactions according to the topological parameters of ∇^2^*ρ*(*rc*) > 0 and *H*(*rc*) > 0. Moreover, the structure is additionally stabilized by several C-H⋯O hydrogen bonds between the PVP-tetramer and the (TiO_2_)_10_ cluster (*d*_H⋯O_ = 2.510 to 3.633 Å and *E*_H⋯O_ = −2.06 to −0.11 kcal mol^−1^). In addition, the Ca^2+^ ion is bound in between the PVP and TiO_2_ surface and is coordinated by two oxygen atoms from the PVP-tetramer and three oxygen atoms from the (TiO_2_)_10_ cluster, as shown in Figure 4a [PVP-Ca-TiO_2_-I structure: ∆G*_INT_ = −63.82 kcal mol^−1^]. Two Ca^2+^-O_carbonyl_ bonds have a bond length of 2.292 and 2.289 Å, and an energy of −12.64 and −13.59 kcal mol^−1^, respectively. The other three coordinate Ca^2+^-O_(TiO2)10_ bonds (*d*_O⋯Ca_ = 2.465, 2.394 and 2.468 Å; *E*_O⋯Ca_ = −8.79, −10.98 and −8.94 kcal mol^−1^), through which the Ca^2+^ ions are bound to the (TiO_2_)_10_ cluster, which additionally strengthen the adhesion of the PVP layer to the TiO_2_ surface, play an important role in the stability of the (PVP+Ca^2+^) coating. All Ca^2+^-O bonds formed can be considered as ionic types of interactions according to the positive values of ∇^2^*ρ*(*rc*) and *H*(*rc*) from the topological electron density analysis. The advantages of forming a PVP-Ca-TiO_2_-I structure should be emphasized compared to forming a PVP-Ca-TiO_2_-II structure, as shown in Figure 4b, with higher values of Gibbs free energy of interactions for PVP-Ca-TiO_2_-I, ∆G*_INT_ = −63.82 kcal mol^−1^, than PVP-Ca-TiO_2_-II structure, ∆G*_INT_ = −57.32 kcal mol^−1^. 

In the less stable PVP-Ca-TiO_2_-II structure (Figure 4b), the PVP tetramer is linked similarly to the PVP-Ca-TiO_2_-I structure via a coordinate O_carbonyl_-Ti bond (*d*_O⋯Ti_ = 2.049 Å; *E*_O⋯Ti_ = −28.80 kcal mol^−1^) to the TiO_2_ surface, which is also supported by several C-H⋯O hydrogen bonds (ranging from 2.296 to 2.858 Å and −3.40 to −0.89 kcal mol^−1^). It should be noted that the O_carbonyl_-Ti bond in the PVP-Ca-TiO_2_-II structure is weaker than that in the PVP-Ca-TiO_2_-I structure, which may be one of the main reasons for the weaker bonding of the PVP layer to the TiO_2_ surface compared to that obtained in the PVP-Ca-TiO_2_-I structure. Moreover, in contrast to the previous structure, the Ca^2+^ ions are now bound on the top of the PVP layer (*d*_O⋯Ca_ = 2.243, 2.226 and 2.225 Å; *E*_O⋯Ca_ = −15.60, −16.17 and –16.57 kcal mol^−1^, respectively), so they are not involved in the binding of the PVP to the TiO_2_ surface, which ultimately leads to the less stable structure.

In summary, the process of formation of (PVP+Ca^2+^) fibers on the TiO_2_ surface is a thermodynamically favorable process, mainly due the PVP-Ca-TiO_2_-I structure, in which the fibers are formed by a strong coordinate O_carbonyl_-Ti bond between the pyrrolidone ring of the PVP and the TiO_2_ surface. Several hydrogen C-H---O bonds are present and Ca^2+^ ions are located between the PVP layer and the TiO_2_ surface. The strongly coordinated Ca^2+^ ions by both the PVP layer and the TiO_2_ surface enhance the adhesion of the (PVP+Ca^2+^) fibers to the TiO_2_ surface. This is consistent with the thermodynamic analysis. The bonding is more exergonic when the Ca^2+^ ions interact simultaneously with the TiO_2_ surface and the PVP polymer, as is the case for the PVP-Ca-TiO_2_-I structure, compared to the PVP-Ca-TiO_2_-II structure, where the Ca^2+^ ions are bound on the top of the PVP layer. However, both the PVP-Ca-TiO_2_ structures described above would compete energetically in the formation of the composite fibers. Consequently, the complex structure of these fibers could influence the release of Ca^2+^ ions. While in the less stable PVP-Ca-TiO_2_-II structure the Ca^2+^ ions can leave the surface very easily, in the more stable PVP-Ca-TiO_2_-I structure the Ca^2+^ ions are firmly coordinated between the PVP layer and the TiO_2_ surface, which suppresses their release.

The dissolution of the fibers and the possibility of the release of Ca**^2+^** ions were investigated experimentally in the model electrolyte of the artificial saliva using electrochemical impedance spectroscopy (detailed in the next section).

### 2.3. Monitoring Processes at the Ti/Ti Oxide/(PVP+Ca^2+^) Fibers/Artificial Saliva Interface

**Figure 5:**
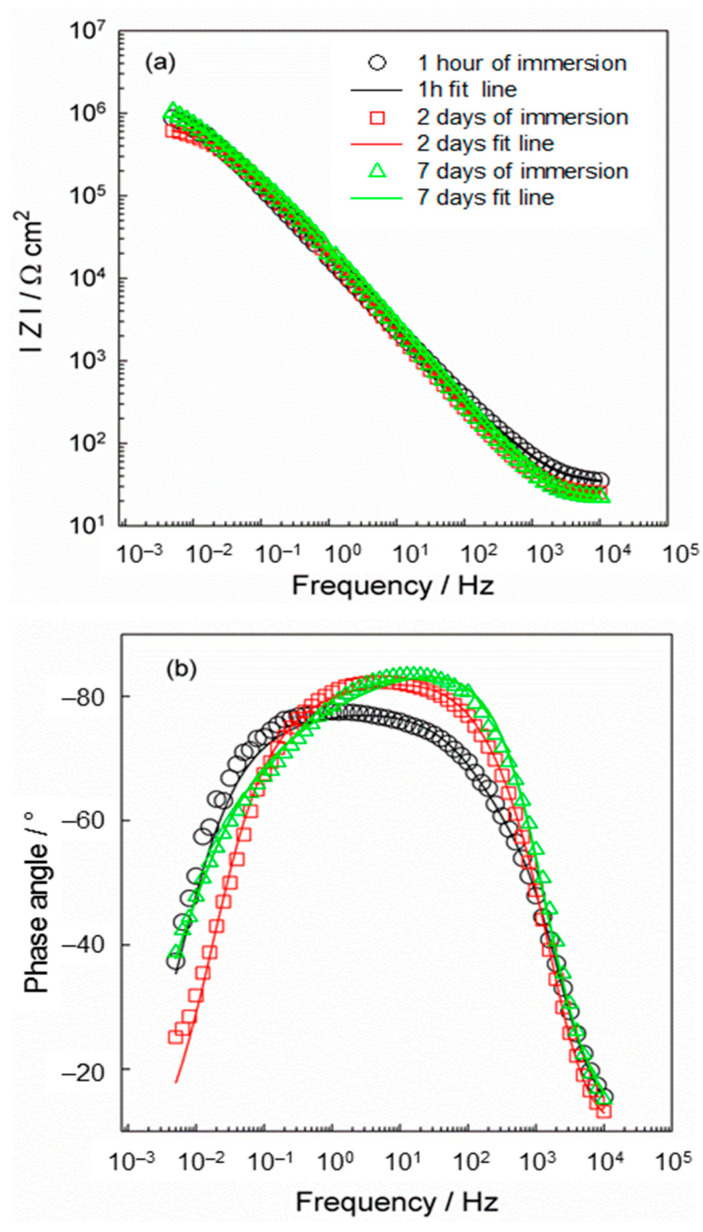
The EIS plots in the form of (**a**) magnitude vs. log *f,* (**b**) phase angle vs. log *f* recorded on the [Ti/Ti oxide/(PVP+Ca^2+^) fibers] sample at open circuit potential after 1 h, 2 days and 7 days of immersion in artificial saliva, pH = 6.8.

The dissolution of the (PVP+Ca^2+^) fibers on the Ti/Ti oxide was monitored during a period of 7 days of immersion in artificial saliva using the electrochemical impedance spectroscopy (EIS) method, and the results are shown in Figure 5.

The EIS spectra were modeled with an electrical equivalent circuit (EEC) with two time constants, which is listed together with the results in Table 1. Due to the microscopic inhomogeneities of the investigated system, a constant phase element (CPE) was used instead of a capacitor (*C*) [56,57]. The impedance of CPE can be expressed as *Z*_CPE_ = [*Q*(jω)^n^]^−1^, where *Q* and n are the parameters associated with CPE. For the CPE exponent n = 1, the frequency–independent CPE parameter *Q* represents the capacitance of the interface. For n ≠ 1, the system shows a behavior that is attributed to the heterogeneity of the surface, the presence of surface films or to the continuously distributed time constants for charge transfer reactions [57]. *R*_el_ is the electrolyte resistance. The high/medium frequency time constant (*R*_1_CPE_1_) is related to the resistance and capacitance of the (PVP+Ca^2+^) fibers, while the low frequency time constant (*R*_2_CPE_2_) represents the resistance and capacitance of the thermally prepared oxide film.

As can be seen in Figure 5a, the EIS responses were altered over a period of 7 days. During the first 2 days of immersion of the modified titanium in artificial saliva, a decrease in the I*Z*I vs. log *f* value can be observed, indicating a decrease in the overall resistivity value of the system, as shown in Table 1 (both resistance components decrease). The dependence of the phase angle on log *f* also shows that the width of the response decreases after 2 days of immersion compared to the response after 1 h of immersion. From all this it can be concluded that the porous (PVP+Ca^2+^) fiber network obviously dissolves after the immersion of the modified Ti sample in saliva. This is to be expected, as PVP is a hydrophilic polymer and begins to dissolve on contact with water. Due to its hydrophilic nature, the fiber network dissolves, leaving a significant portion of the Ti/Ti oxide surface accessible to saliva.

After the initial decrease in the system resistance due to the beginning of the dissolution of the fibers, two processes most likely take place simultaneously: (i) the further dissolution of the (PVP+Ca^2+^) fibers, and also (ii) the growth (thickening) of the thermally prepared oxide film on the surface. This is confirmed by the renewed increase in the resistance of the investigated system after 7 days of immersion time, which can be seen from the I*Z*I vs. log *f* dependence, which increases again. The modeled data also confirm this. The total resistance value of the system increases due to the thickening of the oxide film. As can be seen from Table 1, not only does the resistance component *R*_1_ increase, but the values of the second time constant also change considerably. The resistance *R*_2_ increases and the value of the capacitance decreases, i.e., the exponent n_2_ and CPE_2_ decrease. In general, a decrease in the capacitance value indicates an increase in the layer thickness. 

After the dissolution of the fiber network, the oxide film remained on the surface and the EIS response after 7 days of immersion is associated with the thickening and further growth of the oxide film. It is known that compact and protective film is formed next to the Ti surface, while the amorphous part of the film is located at the outer boundary, the oxide film/electrolyte interface [58,59]. Therefore, the parameters of the high/medium frequency response (*R*_1_CPE_1_) can be attributed to the growth of the amorphous part of the oxide film that forms at the outer oxide film/electrolyte interface. At low frequency values, the more compact part of the film dominates at the Ti/Ti oxide interface. Such a structure is confirmed by the values *R*_1_ and *R*_2_. The resistance value of the amorphous part of the oxide film, *R*_1_, is significantly lower (Ω cm^2^) than the value of *R*_2_ (MΩ cm^2^), which indicates the protective part of the oxide film.

The EIS results indicate a gradual dissolution of the fiber network accompanied by the release of Ca^2+^ ions, which is consistent with the DFT results. The modeling of the EIS data have shown that the surface of the modified Ti sample is partially covered with the fibers after 2 days of immersion, while the oxide dominates after 7 days of immersion. This behavior can be explained by the complex structure of the composite fibers, which was determined by DFT calculations. As shown, the less stable PVP-Ca-TiO_2_-II structure (∆G*_INT_ = −57.32 kcal mol^−1^; Figure 4b), in which the Ca^2+^ ions are bound on the top of the PVP layer, apparently contributes to the dissolution of the composite fibers immediately after the modified Ti is immersed in the artificial saliva. In the next step, the more stable PVP-Ca-TiO_2_-I structure (∆G*_INT_ = −63.82 kcal mol^−1^; Figure 4a), in which the Ca^2+^ ions are firmly coordinated between the PVP layer and the TiO_2_ surface, would further dissolve.

**Figure 6:**
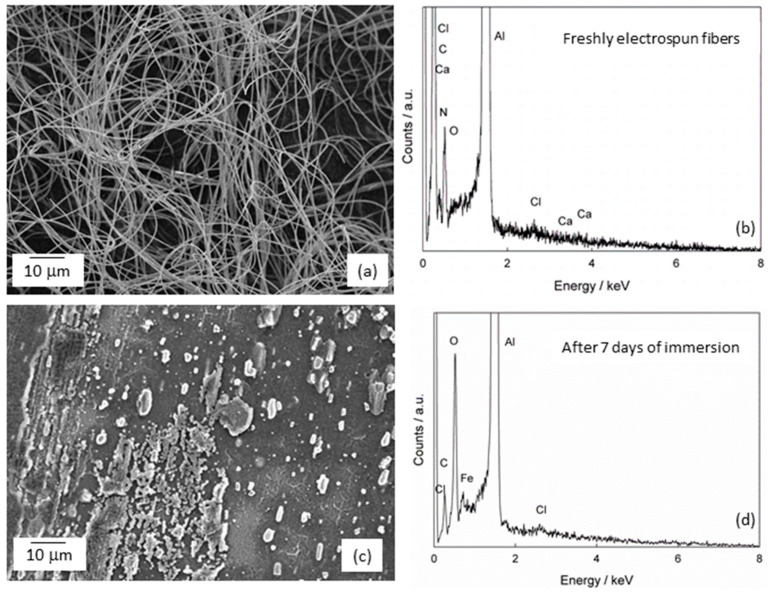
The SEM images and corresponding EDS spectra of (**a**,**b**) freshly electrospun (PVP+Ca^2+^) fibers on Al foil; (**c**,**d**) the Al foil surface with residual fibers after 7 days of immersion in artificial saliva, pH = 6.8. Fe, visible in the spectrum (**d**), originates from Al foil.

For an additional confirmation of the solubility of the (PVP+Ca^2+^) fibers, the same fibers were electrospun, but on an Al foil (Section 3.3). After a short drying period, the foil was immersed in artificial saliva for 7 days. The results, determined by SEM and EDS, are shown in Figure 6. 

The freshly electrospun fibers are homogeneous and contain PVP and calcium (Figure 6a,b). After a 7-day immersion in artificial saliva, the appearance of the surface shows that the structure of the fibers has been destroyed and the fibers have dissolved significantly (Figure 6c,d). The EDS spectrum (Figure 6d), in which both the element N (from PVP) and Ca are absent, confirms that the dissolution of the composite fibers and the release of Ca^2+^ ions have taken place. Cl ions are visible in the spectrum, most likely due to the interaction with the Al foil.

All results indicate that the complex structure of the fibers is a key factor for the dissolution and release of Ca^2+^ ions. Further detailed studies on the kinetics of Ca^2+^ ion release are required. However, it can be concluded that the dissolution of the composite fibers, which can be observed in a short time, could create such an environment around the Ti that favors the formation of new bone, the first signs of which may appear within 4 days after implantation [60]. To initiate the complicated process of new bone formation, Ca^2+^ ions play an important role in stimulating the movement of pre-osteoblasts to the site of bone resorption and their maturation into cells that form new bone [61,62].

## 3. Materials and Methods

### 3.1. Chemicals, Solutions and Materials

Polyvinylpyrrolidone (*M*w = 1,300,000; Alfa Aesar^®^, Karlsruhe, Germany), acetone (p.a.; Gram-mol^®^, Zagreb, Croatia), ethanol absolute (p.a.; Gram-mol^®^, Zagreb, Croatia) and CaCl_2_ (p.a.; Kemika^®^, Zagreb, Croatia) were used as received. A PVP solution, prepared by dissolving 3 g PVP in 50 mL ethanol absolute and 5 mL Milli-Q water (Milli-Q^®^ Direct Water Purification System, Merck^®^, Darmstadt, Germany), served as the basis for the preparation of the fibers. The mixture was stirred for 7 h at 350 rpm and 70 °C. CaCl_2_ was added to this PVP solution and stirred ultrasonically for 10 min to obtain a 10^−2^ mol dm^−3^ solution of CaCl_2_ in PVP.

The Fusayama artificial saliva [63] was used as a model solution to monitor the processes on the Ti/Ti oxide/fibers/saliva interface. The solution was prepared from p.a. grade chemicals and Milli-Q water [0.4 g dm^−3^ NaCl (Kemika^®^, Zagreb, Croatia), 0.4 g dm^−3^ KCl (Kemika^®^, Zagreb, Croatia), 0.6 g dm^−3^ CaCl_2_·2H_2_O (Kemika^®^, Zagreb, Croatia), 0.58 g dm^−3^ Na_2_HPO_4_·2H_2_O (Kemika^®^, Zagreb, Croatia) and 1 g dm^−3^ urea (Kemika^®^, Zagreb, Croatia)].

**Figure 7:**
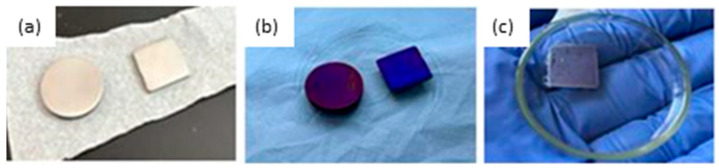
The photographs of (**a**) the freshly abraded and degreased surface of the Ti samples; (**b**) the thermally generated oxide film on the Ti (Ti/Ti oxide); and (**c**) the (PVP+Ca^2+^) fibers on the Ti/Ti oxide sample.

Titanium (Ti, 99.9%, Alfa Aesar^®^, Karlsruhe, Germany) in the form of disks (*A* = 1.77 cm^2^) and plates (*A* = 1.69 cm^2^) was used as a substrate for the modification. Each sample was abraded with 200, 500 and 600 grit SiC emery paper, ultrasonically cleaned with acetone, Mili-Q^®^ water and absolute ethanol (5 min per solvent) and finally dried with nitrogen gas (99.999%, Messer^®^, Bad Soden, Germany); Figure 7a. To create the oxide film on the surface of the samples, the samples were thermally treated in an oven (Instrumentaria, Zagreb, Croatia) from room temperature to 450 °C (10 °C/min) and left for 2 h (Figure 7b). The samples prepared in this way are labeled as Ti/Ti oxide samples.

### 3.2. Preparation of the (PVP+Ca^2+^) Fibers on the Ti/Ti Oxide Surface—[Ti/Ti Oxide/(PVP+Ca^2+^) Fibers]

For the synthesis of (PVP+Ca^2+^) fibers, the Starter Kit-Random device (Linari Engineering s.r.l., Pisa, Italy) was used under the following conditions: voltage 10.02 kV, flow rate 1 mL h^−1^, time 3 min, distance between metal needle and Ti plate 10 cm, metal needle diameter 1 mm, room temperature, *T* = 23 ± 2 °C and relative humidity ~60%. The (PVP+Ca^2+^) fibers were electrospun on the Ti/Ti oxide surface from the 10^−2^ mol dm^−3^ solution of CaCl_2_ in PVP. The Ti/Ti oxide plate was attached to the aluminum collector with a carbon tape. After 3 min of collection, a white deposit formed on the surface of the Ti/Ti oxide plate; Figure 7c. This sample was labeled [Ti/Ti oxide/(PVP+Ca^2+^) fibers].

For some analyses, fibers of bare PVP and (PVP+Ca^2+^) fibers were electrospun on the aluminum foil under the same conditions as for the (PVP+Ca^2+^) fibers on the Ti. 

### 3.3. Preliminary Study of the Dissolution of the (PVP+Ca^2+^) Fibres

In order to quickly evaluate the solubility of the composite (PVP+Ca^2+^) fibers, the fibers were electrospun on aluminum foil under the same conditions as described in Section 3.2 due to the simplicity of the experiment. The electrospun (PVP+Ca^2+^) fibers on the foil were immersed in artificial saliva for 7 days. After drying in air, the foil with the remaining fibers was examined by the SEM and EDS methods.

### 3.4. Characterization of Ti Samples

The morphology of the (PVP+Ca^2+^) fibers was investigated using a thermal field emission scanning electron microscope (FE-SEM, model JSM-7000F, Jeol, Ltd., Tokyo, Japan) in conjunction with Oxford Instruments’ EDS/INCA 350 energy dispersive X-ray analyzer (High Wycombe, UK) for elemental analysis.

The chemical composition of the Ti samples was analyzed using PerkinElmer’s Frontier MIR spectrometer (Waltham, MA, USA) for Fourier transform infrared measurements in attenuated total reflectance mode (ATR-FTIR). Measurements were performed in the wavenumber range between 4000 and 400 cm^−1^ at a resolution of 4 cm^−1^ and 20 scans per measurement.

The processes at the Ti/Ti oxide/(PVP+Ca^2+^) fibers/Fusayama artificial saliva interface were investigated at open circuit potential (*E*_OCP_) after 1 h, 2 and 7 days of immersion by electrochemical impedance spectroscopy (EIS). The period of 7 days was chosen as this is an initial period in the process of osseointegration of dental implants. EIS measurements were performed in a three-electrode cell in the frequency range of 10^5^ to 5 × 10^−3^ Hz with an *ac* voltage amplitude of ±5 mV using the Solartron 1287 potentiostat/galvanostat with Solartron FRA 1260, controlled by ZPlot^®^ software (v. 3.5e, Scribner Associates Inc., Southern Pines, Moore, NC, USA). The Ag/AgCl, 3 mol dm^−3^ KCl electrode (*E* = 0.210 V vs. standard hydrogen electrode, SHE) served as the reference electrode, with the two graphite rods as the counter electrode, while the Ti samples served as the working electrode. The experimental data were analyzed with complex nonlinear least squares analysis (CNLS) using ZView^®^ software (v. 3.5e, Scribner Associates Inc., Southern Pines, Moore, NC, USA) with χ^2^ values less than 5 × 10^−3^.

### 3.5. Quantum Chemical Calculations

Density functional theory (DFT) quantum chemical calculations have been carried out, employing the M06 functional developed by Truhlar’s group [64,65,66]. The 6-31+G(d,p) + LANL2DZ basis set was employed for geometry optimization. Pople’s 6-31+G(d,p) double-ξ basis set was chosen for the H, C, O, N, and Ca atoms, and the LANL2DZ basis set was chosen for the transition metal (Ti) atoms [67]. The vibrational frequency analysis was performed under the harmonic oscillator approximation and was used to check that all calculated structures are the true minima on the potential energy surface. In addition, the thermal correction to the Gibbs free energy was derived from the same vibrational analysis. The final energy of the considered systems was calculated utilizing a highly flexible 6-311++G(2df,2pd) basis set for H, C, O, N, and Ca atoms, while the same LANL2DZ ECP type basis set was used for the titanium atoms. To model the solvent effects the polarizable continuum solvation model SMD (solvation model based on density) [68] was used. The ethanol solvent is represented by a dielectric constant of ε = 24.852. All calculations were performed with the program package Gaussian 09 (revision D.01) [69].

The topological analysis of the charge density distribution was performed by means of Bader’s quantum theory of atoms in molecules (QTAIM) [70] using the AIMALL [71] program package and utilizing the SMD/M06/6-31+G(d,p) + LANL2DZ wave function obtained from the optimization.

The Gibbs free energy of the interaction, ∆G*_INT_, was calculated according to the formula ∆G*_INT_ = G*_AB_ − G*_A_ − G*_B_, where G*_AB_ is the total free energy of the resulting AB structure, and G*_A_ and G*_B_ are the total free energies of the associating units A and B, respectively. A detailed description of the computational modeling can be found in the Supplementary Materials.

## 4. Conclusions

The composite fibers of polyvinylpyrrolidone (PVP) and Ca^2+^ ions were successfully prepared by electrospinning on the titanium oxide surface. The main objective was to use the composite fibers as a biodegradable coating, with the PVP fibers acting as a temporary carrier of Ca^2+^ ions needed for the growth of bone cells around the titanium once implanted.

The ATR-FTIR measurements of the prepared (PVP+Ca^2+^) fibers revealed that there is an interaction between the carbonyl oxygen of PVP and the Ca^2+^ ions, as evidenced by the band shift of the carbonyl group from 1663 cm^−1^ (bare PVP fibers) to 1660 cm^−1^ (PVP+Ca^2+^ fibers). The spectrum of the [Ti/Ti oxide/(PVP+Ca^2+^) fibers] sample is dominated by bands originating from the PVP, while the band assigned to the oxide film is shifted compared to the bare Ti/Ti oxide sample, pointing to the interaction between the oxide and the composite fibers. All this confirms the successful formation of the (PVP+Ca^2+^) fibers on the Ti/Ti oxide surface.

The SEM results show that the composite (PVP+Ca^2+^) fibers form a porous network on the Ti/Ti oxide surface, in which the fibers are partially bonded together. Such a morphology is most likely a consequence of the weak conductivity of the titanium substrate.

The DFT results reveal the binding of Ca^2+^ ions to the PVP fibers via the carbonyl oxygen atoms of the pyrrolidone ring of the PVP molecule. The (PVP+Ca^2+^) fibers are most likely bound to the Ti/Ti oxide surface by two thermodynamically competing structures. Both structures involve the formation of a coordinate O_carbonyl_-Ti bond formed between the pyrrolidone ring of the PVP molecule and the Ti/Ti oxide surface, supported by several hydrogen C-H⋯O bonds. While in the thermodynamically more stable structure the Ca^2+^ ions interact simultaneously with the TiO_2_ surface and the PVP molecule (∆G*_INT_ = −63.82 kcal mol^−1^), in the less stable structure the Ca^2+^ ions are bound to the top of the PVP layer (∆G*_INT_ = −57.32 kcal mol^−1^).

This complex structure of the composite fibers on the Ti/Ti oxide surface is directly related to the dissolution of the fibers after the immersion of the modified Ti in artificial saliva for 7 days, which was monitored by EIS. Initially, the fibers that are only weakly bound to the Ti/Ti oxide surface (Ca^2+^ on the top of the PVP) are removed/dissolved, while the fibers that are more strongly bound to the surface (Ca^2+^ between the PVP and Ti/Ti oxide) are responsible for further dissolution. After a 7-day immersion in saliva, an oxide film dominates on the Ti surface. It continues to grow and thicken, which, according to the EIS results, has a positive influence on the stability and protective properties of titanium in artificial saliva.

When the PVP fiber component dissolves, Ca^2+^ ions are released, which create the necessary ionic environment in the vicinity of the titanium for the bone-forming cells (osteoblasts) to work effectively. The release of Ca^2+^ in a short time could be an advantage for the complex process of new bone growth around the implant. The Ca^2+^ ions influence cellular activity and tissue formation processes, such as the proliferation, differentiation and mineralization of the matrix. 

## Figures and Tables

**Table 1:** The impedance parameters calculated from EIS data (Figure 5) using an equivalent electrical circuit (shown below) for the [Ti/Ti oxide/(PVP+Ca^2+^) fibers] sample.

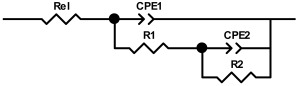							
Immersion Time	*R*_el_/ Ω cm^2^	CPE_1_·10^6^/ Ω^−1^ cm^−2^ s^n1^	n_1_	*R*_1_/ Ω cm^2^	CPE_2_·10^6^/ Ω^−1^ cm^−2^ s^n2^	n_2_	*R*_2_/ ΜΩ cm^2^
1 h	35	5.13	0.929	319	6.71	0.796	1.42
2 days	26	4.68	1	189	6.15	0.809	0.69
7 days	31	4.40	1	536	5.30	0.665	1.95

## Data Availability

The data presented in this study are available on request from the corresponding author.

## Supplementary Materials

The following supporting information can be downloaded at: https://www.mdpi.com/article/10.3390/molecules29174181/s1. It contains: Computational modeling, Figure S1: Optimized structures of the selected systems (bond distances in Å, bond energies in kcal mol^−1^), Table S1: Formation of PVP–Ca and PVP–Ca–TiO_2_. Standard state (1M) Gibbs free energy of interaction computed at the M06/6-311++G(2df,2pd) + LANL2DZ// M06/6-31+G(d,p) + LANL2DZ level of theory (in kcal mol^−1^) in the ethanol solution by using SMD solvation model (formation of the PVP–Ca) and in the gas phase (formation of PVP–Ca–TiO_2_), Table S2: Total electronic energy, *E*^Tot^_soln_, obtained at the SMD/M06/6-311++G(2df,2pd) + LANL2DZ//SMD/M06/6-31+G(d,p) + LANL2DZ level of theory, thermal correction to the Gibbs free energy, Δ*G^*^*_VRT,soln_, obtained at the SMD/M06/6-31+G(d,p) + LANL2DZ level of theory, and total free energy, *G^*^*_X_, (*G^*^*_X_ = *E*^Tot^_soln_ + Δ*G^*^*_VRT,soln_) in ethanol media of the investigated species (all energies in hartree) related to the formation of PVP–Ca, Table S3: Total electronic energy, *E*^Tot^, obtained at the M06/6-311++G(2df,2pd) + LANL2DZ//SMD/M06/6-31+G(d,p) + LANL2DZ level of theory, thermal correction to the Gibbs free energy, Δ*G^*^*_VRT_, obtained at the M06/6-31+G(d,p) + LANL2DZ level of theory, and total free energy, *G^*^*_X_, (*G^*^*_X_ = *E*^Tot^ + Δ*G^*^*_VRT_) in the gas phase of the investigated species (all energies in hartree) related to the formation of PVP–Ca–TiO_2_, Table S4: Bond lengths (d), energies (*E*) and QTAIM properties of the selected bonds in the investigated systems, Cartesian coordinates of the calculated systems. References [72,73,74,75,76,77,78,79,80,81] are cited in the Supplementary Materials.
