# Supplementary material for: Electrospun PVP Fibers as Carriers of Ca2+ Ions to Improve the Osteoinductivity of Titanium-Based Dental Implants
Source: Molecules. 2024 Sep 3;29(17):4181. doi: 10.3390/molecules29174181 (PMC11397674; doi:10.3390/molecules29174181)
Supplement: Supplementary file 1 [file molecules-29-04181-s001.zip › molecules-3104778-supplementary.pdf]

Supporting Information

for

The electrospun PVP fibers as carriers of Ca^{2+} ions to improve the osteoinductivity of titanium-based dental implants

Janina Roknić[1], Ines Despotović[2,*], Jozefina Katić[1], and Željka Petrović [3,*]

Address:

[1] Department of Electrochemistry, Faculty of Chemical Engineering and Technology, University of Zagreb, Marulićev trg 19, 10000 Zagreb, Croatia; roknic57@gmail.com; jkatic@fkit.unizg.hr

[2] Division of Physical Chemistry, Ruđer Bošković Institute, Bijenička cesta 54, 10002 Zagreb, Croatia

[3] Division of Materials Chemistry, Ruđer Bošković Institute, Bijenička cesta 54, 10002 Zagreb, Croatia

*Correspondence: zeljka.petrovic@irb.hr (Ž.P.); ines.despotovic@irb.hr (I.D.)

Computational modeling

All calculations were performed by means of quantum chemical calculations at the density functional theory (DFT) level using the Gaussian 09 program (revision D.01) [69].

The M06 functional designed by the Truhlar's group, which provides very accurate thermodynamic parameters, and is particularly successful in nonbonding interactions treatment, has been selected [64–66]. The 6-31+G(d,p) + LANL2DZ mixed basis set has been utilized. The Pople's 6-31+G(d,p) double-ξ basis set was chosen for O, H, C, N, Ca atoms and the LANL2DZ basis (LANL2 pseudopotential for inner electrons and its associated double-ξ basis set (DZ)) was used for the transition-metal (Ti) atoms [67]. This gave rise to the M06/6-31+G(d,p) + LANL2DZ model utilized for geometry optimization which has been frequently used for studies of transition-metal containing systems. The geometric structures of the molecules were optimized by minimizing energies with respect to the geometrical parameters without imposing any molecular symmetry constraints and using a tight convergence condition. The Berny algorithm using redundant internal coordinates was employed. Frequency calculations were made under the harmonic approximation on all the optimized structures at the same level of theory with no scaling in order to confirm that the structures correspond to the true minima, meaning that no imaginary frequencies were present, as well as to extract thermal Gibbs free energy corrections. The final single point energies were obtained using a highly flexible 6-311++G(2df,2pd) basis set for the O, H, C, N, Ca atoms, while the same LANL2DZ ECP type basis set for titanium atoms was employed.

The self- consistent field (SCF) calculations were conducted under a tight condition imposing the threshold value of 10^{-8} hartree to total energy difference during the iteration process. The integration grid was set to FineGrid having 75 radial shells and 302 angular points per shell. The 2-electron integral accuracy was set to 10^{-13}. The FoFCou algorithm with NoSymm option was utilized. All the geometry optimizations, frequency calculations and single point energy evaluations were performed by taking solvent effects into account. To evaluate the bulk solvent effects (ethanol, ε = 24.852 at 25°), the implicit SMD polarizable continuum solvation model [68] has been employed. It represents a very practical approach to simulate the solvation environment and determine the effect of the medium on the structure and stability of solutes in a solution.

The conformational space was manually sampled for the PVP-Ca and PVP–Ca–TiO_2 taking into account various interaction sites between PVP and Ca^{2+} as well as between PVP–Ca species and TiO_2 surface. The $(TiO_2)_{10}$ clusters were employed as proposed by Allard et al. [72] and Qu and Kroes [55]. The PVP was modelled by the short PVP-tetramer chains.

The interaction Gibbs free energies, ΔG^{*}_{INT} were computed as the difference between the total free energy (G^{*}_{AB}) of the PVP-Ca (PVP–Ca–TiO_2) structure and the sum of the total free energies (G^{*}_{A} + G^{*}_{B}) of the associating units A and B (PVP, Ca^{2+} ion; PVP–Ca, $(TiO_2)_{10}$ cluster) using the supramolecular approach:

$$\Delta G^{*}_{INT,AB} = G^{*}_{AB} - G^{*}_{A} - G^{*}_{B} \quad (1)$$

The species total free energy in the gas phase was calculated using the expression:

$$G^{*}_{X} = E^{Tot} + \Delta G^{*}_{VRT} \quad (2)$$

where E^{Tot} corresponds to the basic energy of a density functional theory calculation, while ΔG^{*}_{VRT} encompasses vibrational, rotational and translational contribution to the free energy.

The species total free energy in the liquid was calculated using the expression:

$$G^*_X = E^{Tot}_{soln} + \Delta G^*_{VRT,soln} \quad (3)$$

where E^{Tot}_{soln} corresponds to the basic energy of a density functional theory calculation using the SMD model, while $\Delta G^*_{VRT,soln}$ encompasses vibrational, rotational and translational contribution to the solution free energy, being computed by applying the ideal gas partition functions to the frequencies calculated in the dielectric medium and the 1M standard state.

A more negative value of the binding energy implied the more stable formed species. No BSSE correction of binding energies has been applied.

The topological analysis of the charge density distribution using the Bader's quantum theory of atoms in molecules (QTAIM) [70] was performed by employing AIMALL software package [71] using the SMD/M06/6-31+G(d,p) + LANL2DZ wave function obtained from optimization. Within the QTAIM analysis the electron density was analysed for two major characteristics: (a) the existence of critical points (CPs), where electron density exhibits maximum, minimum, or a saddle point in space, and (b) for the bond paths [73], the maximum electron density line connecting two interacting atoms in the energetic minimum structure (the two atoms are bonded). The point of the electron density minimum value along that line called the bond critical point (BCP) and values of topological parameters, like electron density $\rho(r_c)$, Laplacian $\nabla^2\rho(r_c)$, electronic kinetic energy $G(r_c)$, electronic potential energy density $V(r_c)$, total energy density $H(r_c)$ at that point explain interatomic interaction features. $\nabla^2\rho(r_c) < 0$ indicates locally concentrated charge density, while locally depleted charge density is indicated by $\nabla^2\rho(r_c) > 0$. The chemical bond nature can be described qualitatively concerning signs and values of the electron density Laplacian $\nabla^2\rho(r_c)$ and the electron energy density $H(r_c)$ at the bond critical point according to following criteria. The interactions characterized by $\nabla^2\rho(r_c) < 0$ and $H(r_c) < 0$ (shared interaction) are characteristic for weakly polar and nonpolar covalent bonds. On the other hand, $\nabla^2\rho(r_c) > 0$ and $H(r_c) > 0$ (closed shell interactions) point to ionic bonds, weak hydrogen bonds, and van der Waals interactions. The intermediate interactions which include strong hydrogen bonds and most of the coordinate bonds are characterized by $\nabla^2\rho(r_c) > 0$ and $H(r_c) < 0$ [74,75]. A very high negative value of the $\nabla^2\rho(r_c)$ is an indication of a strong covalent bond, while a high positive value corresponds to a strong noncovalent bond. The energies of the coordinate bonds and of other intermolecular hydrogen bonds have been calculated by the Espinosa's equation [76]:

$$E = 0.5\ V(r_c) \quad (3)$$

where E is the bond energy (a.u.), and $V(r_c)$ is potential energy density (a.u.) at the corresponding critical point. The Espinosa's relationship is widely used for the energy estimation of different types of hydrogen [77,78], van der Waals [79], coordinate [80], and homopolar bonds [81].

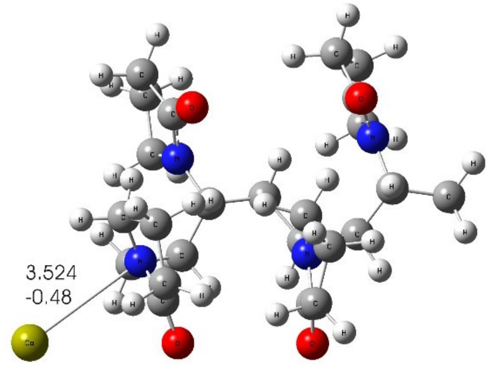

PVP-Ca-I

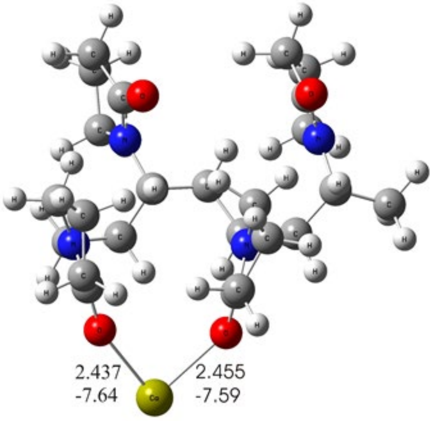

PVP-Ca-II

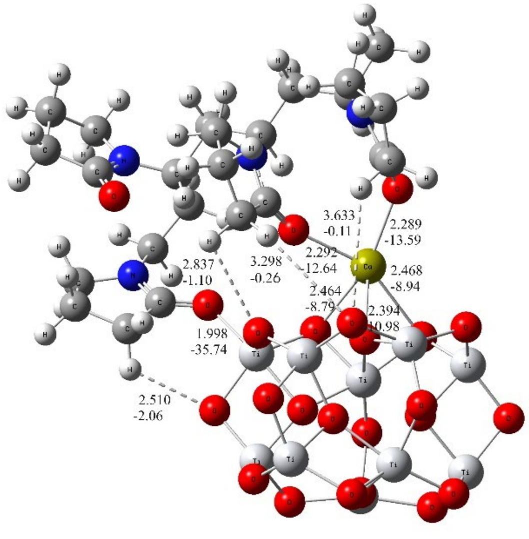

PVP–Ca–TiO_2–I

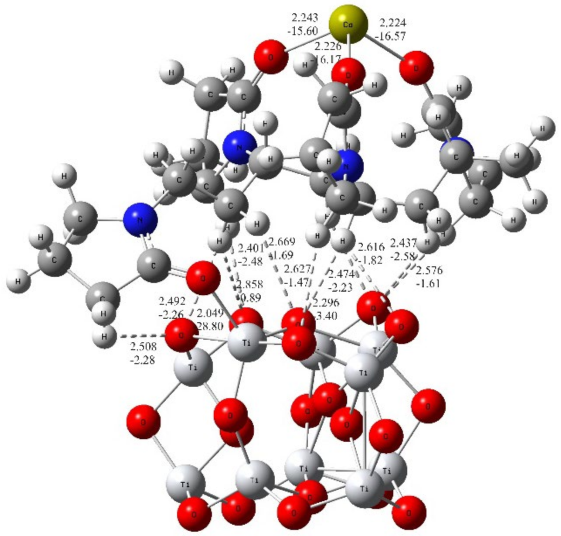

PVP–Ca–TiO_2–II

Figure S1: Optimized structures of the selected systems (bond distances in Å, bond energies in kcal mol^{-1})

Table S1. Formation of PVP–Ca and PVP–Ca–TiO_2. Standard state (1M) Gibbs free energy of interaction computed at the M06/6-311++G(2df,2pd) + LANL2DZ// M06/6-31+G(d,p) + LANL2DZ level of theory (in kcal mol^{-1}) in the ethanol solution by using SMD solvation model (formation of the PVP–Ca) and in the gas phase (formation of PVP–Ca–TiO_2)

Interaction	ΔG^*_{INT}
PVP-tetramer + Ca^{2+} → PVP–Ca–I	9.67
PVP-tetramer + Ca^{2+} → PVP–Ca–II	-2.47
PVP–Ca–II + $(TiO_2)_{10}$ → PVP–Ca–TiO_2–I	-63.82
PVP–Ca–II + $(TiO_2)_{10}$ → PVP–Ca–TiO_2–II	-57.32

Table S2. Total electronic energy, $E^{\mathrm{Tot}}_{\mathrm{soln}}$, obtained at the SMD/M06/6-311++G(2df,2pd) + LANL2DZ//SMD/M06/6-31+G(d,p) + LANL2DZ level of theory, thermal correction to the Gibbs free energy, $\Delta G^{*}_{\mathrm{VRT,soln}}$, obtained at the SMD/M06/6-31+G(d,p) + LANL2DZ level of theory, and total free energy, G^{*}_{X}, ($G^{*}_{\mathrm{X}} = E^{\mathrm{Tot}}_{\mathrm{soln}} + \Delta G^{*}_{\mathrm{VRT,soln}}$) in ethanol media of the investigated species (all energies in hartree) related to the formation of PVP–Ca

Species	$E^{\mathrm{Tot}}_{\mathrm{soln}}$	$\Delta G^{*}_{\mathrm{VRT,soln}}$	G^{*}_{X}
PVP-tetramer	-1456.98999	0.54697	-1456.44302
Ca^{2+}	-677.47988	-0.01521	-677.49509
PVP–Ca–I	-2134.46876	0.54607	-2133.92269
PVP–Ca–II	-2134.49052	0.54848	-2133.94205

Table S3. Total electronic energy, E^{Tot}, obtained at the M06/6-311++G(2df,2pd) + LANL2DZ//SMD/M06/6-31+G(d,p) + LANL2DZ level of theory, thermal correction to the Gibbs free energy, ΔG^{*}_{VRT}, obtained at the M06/6-31+G(d,p) + LANL2DZ level of theory, and total free energy, G^{*}_{X}, ($G^{*}_{X} = E^{Tot} + \Delta G^{*}_{VRT}$) in the gas phase of the investigated species (all energies in hartree) related to the formation of PVP–Ca–TiO_2

Species	E^{Tot}	ΔG^{*}_{VRT}	G^{*}_{X}
PVP–Ca–II_{gas}	-2134.18201	0.55184	-2133.630171
$(TiO_2)_{10}$	-2087.04918	0.02726	-2087.02192
PVP–Ca–TiO_2–I	-4221.35833	0.60453	-4220.75380
PVP–Ca–TiO_2–II	-4221.34394	0.60051	-4220.74343

Table S4. Bond lengths (d), energies (E) and QTAIM properties of the selected bonds in the investigated systems.

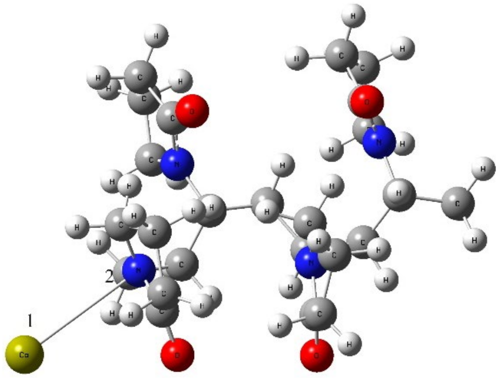

PVP-Ca-I

Bond	d /Å	$\rho(r_c)$/ $e\times a_0^{-3}$	$\nabla^2\rho(r_c)$/ $e\times a_0^{-5}$	$V(r_c)$/au	$G(r_c)$/au	$H(r_c)$/au[a]	E/ kcal mol^{-1} [b]
Ca(1)-N(2)	3.524	2.182×10^{-3}	0.0130	-0.0016	0.0024	0.0008	-0.49

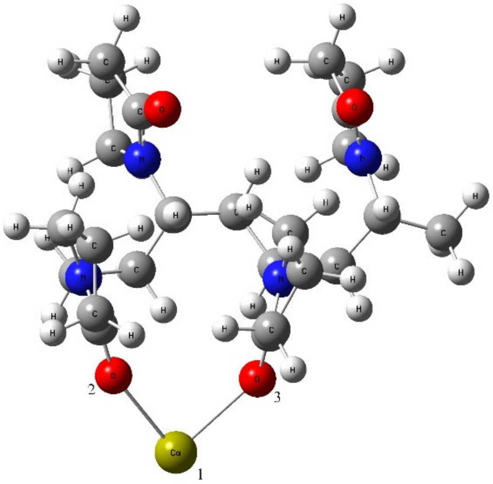

PVP-Ca-II

Bond	d /Å	$\rho(r_c)$/ $e\times a_0^{-3}$	$\nabla^2\rho(r_c)$/ $e\times a_0^{-5}$	$V(r_c)$/au	$G(r_c)$/au	$H(r_c)$/au[a]	E/ kcal mol^{-1}
Ca(1)-O(2)	2.437	2.465×10^{-2}	0.1367	-0.0244	0.0293	0.0049	-7.64
Ca(1)-O(3)	2.455	2.427×10^{-2}	0.1434	-0.0242	0.0300	0.0058	-7.59

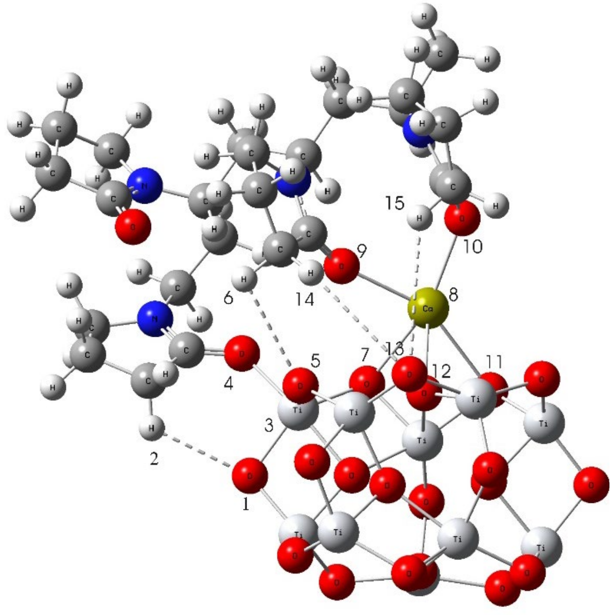

PVP–Ca–TiO_2–I

Bond	d /Å	$\rho(r_c)$/ $e\times a_0^{-3}$	$\nabla^2\rho(r_c)$/ $e\times a_0^{-5}$	$V(r_c)$/au	$G(r_c)$/au	$H(r_c)$/au	E/ kcal mol^{-1}
O(1)-H(2)	2.510	9.735×10^{-3}	0.0344	-0.0066	0.0076	0.0010	-2.06
Ti(3)-O(4)	1.998	8.377×10^{-2}	0.4579	-0.1139	0.1142	0.0003	-35.74
O(5)-H(6)	2.837	5.802×10^{-3}	0.0224	-0.0035	0.0186	0.0021	-1.10
O(7)-Ca(8)	2.464	2.748×10^{-2}	0.1389	-0.0280	0.0314	0.0034	-8.79
O(9)-Ca(8)	2.292	3.583×10^{-2}	0.2247	-0.0403	0.0482	0.0079	-12.64
O(10)-Ca(8)	2.289	3.809×10^{-2}	0.2273	-0.0433	0.0501	0.0068	-13.59
O(11)-Ca(8)	2.468	2.798×10^{-2}	0.1376	-0.0285	0.0315	0.0030	-8.94
O(12)-Ca(8)	2.394	3.284×10^{-2}	0.1699	-0.0350	0.0387	0.0037	-10.98
O(13)-H(14)	3.298	2.352×10^{-3}	0.0073	-0.0008	0.0013	0.0005	-0.26
O(13)-H(15)	3.633	1.005×10^{-3}	0.0037	-0.0003	0.0006	0.0003	-0.11

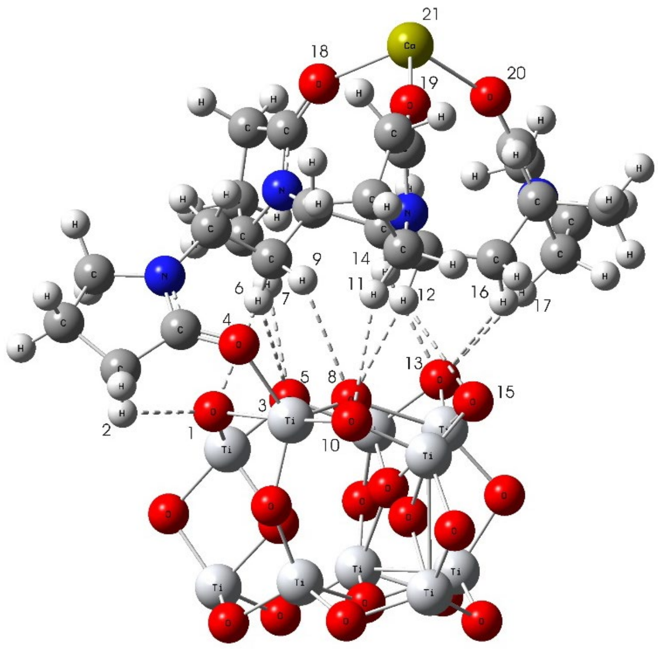

PVP–Ca–TiO_2–II

Bond	d /Å	$\rho(r_c)$/ $e\times a_0^{-3}$	$\nabla^2\rho(r_c)$/ $e\times a_0^{-5}$	$V(r_c)$/au	$G(r_c)$/au	$H(r_c)$/au	E/ kcal mol^{-1}
O(1)-H(2)	2.508	1.067×10^{-2}	0.0389	-0.0073	0.0085	0.0012	-2.28
Ti(3)-O(4)	2.049	7.046×10^{-2}	0.3883	-0.0918	0.0944	0.0026	-28.80
O(5)-H(6)	2.858	4.951×10^{-3}	0.0187	-0.0028	0.0038	0.0009	-0.89
O(5)-H(7)	2.401	1.109×10^{-2}	0.0350	-0.0079	0.0083	0.0004	-2.48
O(8)-H(9)	2.669	8.793×10^{-3}	0.0292	-0.0054	0.0064	0.0010	-1.69
O(8)-H(12)	2.296	1.475×10^{-2}	0.0433	-0.0108	0.0108	0.0000	-3.40
O(10)-H(11)	2.627	7.504×10^{-3}	0.0250	-0.0047	0.0055	0.0008	-1.47
O(13)-H(14)	2.474	1.049×10^{-2}	0.0334	-0.0071	0.0077	0.0006	-2.23
O(13)-H(16)	2.437	1.205×10^{-2}	0.0361	-0.0082	0.0086	0.0004	-2.58
O(13)-H(17)	2.576	7.737×10^{-3}	0.0263	-0.0051	0.0058	0.0007	-1.61
O(15)-H(12)	2.616	8.869×10^{-3}	0.0303	-0.0058	0.0067	0.0009	-1.82
O(18)-Ca(21)	2.243	4.147×10^{-2}	0.2650	-0.0497	0.0580	0.0083	-15.60
O(19)-Ca(21)	2.226	4.202×10^{-2}	0.2792	-0.0515	0.0606	0.0091	-16.17
O(20)-Ca(21)	2.224	4.300×10^{-2}	0.2803	-0.0528	0.0614	0.0086	-16.57

[a]$H(r_c) = V(r_c) + G(r_c)$; [b]$E = 0.5 \times V(r_c)$

Cartesian coordinates of the calculated systems

PVP-tetramer

C	0.245766	0.127014	2.282413
N	0.099481	-0.907337	3.139265
C	0.978264	-2.033135	2.821203
C	2.068709	-1.377483	1.979852
C	1.332759	-0.230140	1.297254
C	-0.946034	-0.977001	4.147713
C	-0.611847	-0.161354	5.390646
O	-0.425887	1.166543	2.294159
H	2.540689	-2.076912	1.284584
H	2.848510	-0.978419	2.645389
H	0.419205	-2.795313	2.255718
H	1.359763	-2.497514	3.737621
H	0.838842	-0.555935	0.369854
H	1.950027	0.639662	1.050971
H	-1.090787	-2.037323	4.390588
H	1.469536	-0.605808	5.281379
H	-1.450391	-0.217975	6.100070
C	1.633567	-2.820772	6.491810
N	0.615649	-1.936883	6.585164
C	-0.523656	-2.449552	7.347214
C	0.053144	-3.672283	8.062120
C	1.216820	-4.099531	7.173688
C	0.691292	-0.575085	6.056858
O	2.712647	-2.614254	5.920184
H	-0.695951	-4.454977	8.209031
H	0.429087	-3.375678	9.049132
H	-1.346415	-2.718689	6.668178
H	-0.897310	-1.688391	8.042566
H	0.905972	-4.807326	6.391757
H	2.060185	-4.547038	7.707899
H	-1.885001	-0.611273	3.712571
H	-0.533315	0.891186	5.090550
C	1.161128	0.351175	7.181219
H	0.348514	2.172059	6.419976
H	2.112069	-0.053661	7.552824
C	1.900495	2.729048	4.587602
N	2.235768	1.989994	5.673706
C	3.502690	1.270795	5.488449
C	4.171425	2.044604	4.355813
C	2.992515	2.587237	3.558710
C	1.315642	1.817675	6.800877
C	1.635802	2.760444	7.966781
O	0.861895	3.389042	4.460927

H	4.845540	1.413670	3.769575
H	4.755754	2.875450	4.770707
H	3.313425	0.224504	5.200011
H	4.090658	1.256366	6.411080
H	2.637281	1.864913	2.806241
H	3.174795	3.535060	3.044115
H	1.660228	3.778020	7.550401
H	0.781210	2.743835	8.661295
H	0.442314	0.296506	8.013172
H	2.349826	4.030656	10.280036
C	3.754867	0.329348	9.442508
N	2.903569	1.368478	9.597403
C	1.876685	1.111990	10.607713
C	2.451074	-0.061234	11.399801
C	3.335396	-0.771541	10.382689
C	2.926830	2.573393	8.765730
C	3.199819	3.800861	9.623956
O	4.691800	0.274838	8.635425
H	1.667565	-0.696953	11.821238
H	3.064593	0.315699	12.227137
H	0.923329	0.845617	10.125803
H	1.705726	2.003058	11.222367
H	2.770395	-1.504278	9.783284
H	4.204055	-1.287470	10.801733
H	3.773144	2.435489	8.080896
H	3.371789	4.675570	8.986438
H	4.088462	3.650899	10.247920

PVP-Ca-I

C	-0.273080	0.252715	0.354800
C	-0.080089	0.054468	1.836977
N	1.242540	-0.091463	2.077428
C	2.049615	-0.011588	0.859716
C	1.109426	0.660436	-0.138867
O	-0.980197	-0.007205	2.684483
C	1.806635	-0.402454	3.392449
C	2.690300	0.743077	3.866426
C	2.585427	-1.719054	3.404073
C	1.814098	-3.042284	3.316466
N	0.952090	-3.239019	4.485760
C	1.408470	-3.852752	5.603470
C	0.292042	-3.901680	6.614609
C	-0.705324	-2.870062	6.100522

C	-0.464428	-2.866236	4.593538
O	2.548439	-4.310789	5.754960
C	1.078045	-3.251083	2.000898
C	0.341241	-4.581817	1.836201
N	-0.241883	-4.612380	0.496441
C	-1.491710	-4.168274	0.233886
C	-1.718428	-4.245222	-1.255435
C	-0.305359	-4.309643	-1.826058
C	0.505459	-4.967930	-0.710035
O	-2.307016	-3.783131	1.082413
C	1.164439	-5.826574	2.124638
C	0.320680	-7.095159	2.200382
N	-0.736196	-6.995942	3.197001
C	-0.526116	-7.099827	4.527026
C	-1.856484	-6.964030	5.229404
C	-2.768693	-6.360123	4.167305
C	-2.152084	-6.838810	2.856070
O	0.569826	-7.313321	5.062278
Ca	-0.702522	3.523208	-10.504208
H	-3.817004	-6.653207	4.268032
H	-2.718444	-5.263206	4.212526
H	-2.567265	-7.805452	2.529209
H	-2.277741	-6.123737	2.036179
H	-2.178684	-7.972290	5.530743
H	-1.759927	-6.369388	6.143830
H	-0.159443	-7.310863	1.237390
H	-0.516998	-4.587036	2.519144
H	1.959028	-5.960520	1.376476
H	-0.235703	-4.857580	-2.769586
H	0.070798	-3.292237	-1.995595
H	0.550280	-6.062187	-0.820651
H	1.534194	-4.593355	-0.652669
H	-2.293305	-5.159135	-1.463616
H	-2.313110	-3.394230	-1.600477
H	0.968376	-7.947823	2.444130
H	1.668606	-5.696788	3.090920
H	2.581143	-3.825044	3.399176
H	0.334227	-2.459247	1.840727
H	-1.745832	-3.101448	6.348562
H	-0.467451	-1.881764	6.514752
H	-1.097633	-3.617674	4.094719
H	-0.660594	-1.899022	4.118991
H	-0.117676	-4.923748	6.603422
H	0.672952	-3.713058	7.622703
H	3.152512	-1.744722	4.345450
H	3.339413	-1.702607	2.601856

H	1.824534	-3.151139	1.197793
H	3.603757	0.822123	3.262262
H	1.315514	0.355727	-1.168660
H	1.221209	1.749733	-0.077355
H	2.337385	-1.021098	0.526599
H	2.967713	0.559402	1.038939
H	-0.588273	-0.717697	-0.061803
H	-1.070810	0.973174	0.152572
H	0.944865	-0.476956	4.069439
H	2.993491	0.580813	4.907048
H	2.154077	1.697410	3.809737

PVP-Ca-II

C	0.388940	-0.327860	2.090485
N	0.198095	-1.341297	2.946546
C	1.046806	-2.498215	2.642805
C	2.161975	-1.881690	1.805845
C	1.466531	-0.714645	1.113220
C	-0.851952	-1.371744	3.955290
C	-0.500924	-0.550487	5.189813
O	-0.256727	0.737189	2.089568
H	2.614205	-2.598110	1.115559
H	2.950517	-1.503697	2.473102
H	0.463141	-3.242204	2.080041
H	1.402905	-2.965722	3.566805
H	0.966792	-1.027307	0.184330
H	2.112523	0.134173	0.868173
H	-1.019641	-2.425413	4.208947
H	1.579508	-1.014658	5.092973
H	-1.341067	-0.589295	5.898475
C	1.711927	-3.237213	6.277813
N	0.701847	-2.343747	6.379616
C	-0.443145	-2.851078	7.136966
C	0.118144	-4.091355	7.834376
C	1.280910	-4.518548	6.944502
C	0.794526	-0.980420	5.862311
O	2.791738	-3.033541	5.707840
H	-0.640214	-4.867858	7.965159
H	0.492221	-3.815599	8.827956
H	-1.270632	-3.100201	6.456049
H	-0.805047	-2.094399	7.843496
H	0.966329	-5.214132	6.153129
H	2.118033	-4.980408	7.476102

H	-1.780816	-0.992047	3.511312
H	-0.404705	0.499526	4.883815
C	1.264826	-0.067541	6.997915
H	0.463205	1.767517	6.259687
H	2.212042	-0.481668	7.367027
C	2.016921	2.296584	4.424136
N	2.352176	1.584749	5.513792
C	3.631667	0.877038	5.340735
C	4.288602	1.632634	4.189529
C	3.104125	2.168216	3.394886
C	1.426540	1.403501	6.639692
C	1.749958	2.330049	7.817041
O	0.958439	2.941063	4.287590
H	4.952478	0.990362	3.604848
H	4.880791	2.467045	4.583962
H	3.447114	-0.176584	5.080325
H	4.218679	0.891964	6.263235
H	2.751728	1.448018	2.638943
H	3.279465	3.120055	2.885027
H	1.793162	3.351695	7.412140
H	0.886096	2.316509	8.499863
H	0.543995	-0.127994	7.827497
H	2.435925	3.562956	10.153647
C	3.802667	-0.157156	9.269029
N	2.977800	0.900309	9.446402
C	1.945516	0.649070	10.452471
C	2.495509	-0.548523	11.225035
C	3.364088	-1.261385	10.196145
C	3.026405	2.116368	8.633694
C	3.296488	3.329424	9.512988
O	4.729081	-0.222331	8.451112
H	1.699163	-1.174210	11.637216
H	3.117140	-0.197538	12.057582
H	0.987210	0.410365	9.965364
H	1.793088	1.532998	11.081965
H	2.784566	-1.975803	9.588594
H	4.223685	-1.799362	10.605995
H	3.883018	1.980025	7.962022
H	3.494731	4.209351	8.890478
H	4.169118	3.159165	10.153807
Ca	-0.899403	3.011705	2.685005

$(TiO_2)_{10}$

Ti	-2.138125	-2.094033	0.072680
Ti	0.596058	0.365227	1.986770
Ti	-2.283614	0.458226	2.153766
Ti	0.744543	2.661823	-0.245060
Ti	3.051423	0.903530	-1.070630
Ti	3.051781	-0.903649	1.071113
Ti	-2.141774	2.095814	-0.070847
Ti	0.596855	-0.366087	-1.988300
Ti	-2.282777	-0.457598	-2.152909
Ti	0.745491	-2.662247	0.243824
O	2.562880	-2.604434	0.823310
O	2.275005	0.175600	-2.539723
O	-3.198215	-1.891552	-1.413881
O	-3.200345	1.892548	1.416641
O	0.973230	2.100728	1.433387
O	-2.805378	-1.159983	1.553680
O	-0.767579	-0.446197	-3.211778
O	-0.996469	-3.465398	0.129700
O	-0.997595	3.464932	-0.130842
O	4.253331	0.000297	0.000432
O	-0.767664	0.446041	3.211038
O	-1.131639	-0.821715	-0.805777
O	0.154975	-1.441039	1.356141
O	0.157705	1.440343	-1.357990
O	1.784055	-0.000885	0.000042
O	-1.134057	0.823351	0.805058
O	-2.805515	1.160413	-1.552253
O	0.974140	-2.101872	-1.434662
O	2.562990	2.604262	-0.822553
O	2.273552	-0.176468	2.539598

PVP-Ca-TiO_2-I

C	-0.244833	0.222170	0.556713
C	0.131667	0.344169	2.007577
N	1.461691	0.294054	2.129586
C	2.140449	0.138216	0.837423
C	1.060545	0.504428	-0.182851
O	-0.681822	0.434966	2.952501
C	2.182714	0.367216	3.406492
C	3.054961	1.614481	3.447701
C	3.004204	-0.891304	3.696192

C	2.213700	-2.167328	3.987644
N	1.662116	-2.778929	2.775098
C	0.346534	-2.981110	2.599889
C	0.130106	-3.884519	1.421173
C	1.474574	-3.867400	0.697735
C	2.476293	-3.522474	1.801202
O	-0.540030	-2.502950	3.330343
C	3.058851	-3.192996	4.748797
C	2.305476	-4.483859	5.119395
N	3.180871	-5.639225	5.033573
C	3.059837	-6.551079	4.020396
C	4.174092	-7.560203	4.168145
C	5.174050	-6.880606	5.103344
C	4.327640	-5.883055	5.904629
O	2.178352	-6.531982	3.165521
C	1.592671	-4.356786	6.464962
C	0.976089	-5.640827	7.025407
N	0.399815	-6.553523	6.038591
C	-0.646486	-6.293047	5.276482
C	-1.047941	-7.490503	4.479684
C	0.089001	-8.488592	4.715851
C	0.787605	-7.971700	5.976431
O	-1.223693	-5.163769	5.269190
Ca	-2.138336	-0.996839	3.986891
O	-3.011839	-2.827253	5.258883
Ti	-3.019413	-4.613093	4.588080
O	-4.913031	-4.133771	4.405170
Ti	-5.638152	-5.751396	4.813669
O	-6.252226	-6.262711	3.129479
Ti	-6.356400	-5.347009	1.588947
O	-5.729252	-3.779212	2.245953
Ti	-7.508512	-2.804907	2.230133
O	-6.494624	-1.626772	1.115980
Ti	-4.823973	-1.210677	1.388005
O	-4.043875	-2.046718	2.829218
Ti	-4.759797	-2.250320	4.906230
O	-4.319405	-0.344777	4.941455
Ti	-5.640285	0.327204	3.873842
O	-6.385135	-1.405154	3.812451
Ti	-8.185491	-0.847273	4.338993
O	-8.509334	-1.859748	5.751735
Ti	-7.479731	-3.492795	5.727091
O	-7.607360	-3.682909	4.036437
O	-5.927310	-2.578087	6.186396
Ti	-3.974381	-3.875793	1.560553
O	-3.822975	-2.506921	0.404957

O	-4.739600	-5.343442	0.739478
O	-2.755380	-4.576164	2.826545
O	-3.895199	-6.177509	5.162928
O	-6.820991	-5.235860	6.070135
O	-8.783228	-1.590784	2.816879
O	-7.315817	0.802032	4.386662
O	-4.902171	0.473460	2.232714
O	-7.909226	-4.287317	1.313888
H	-0.265215	-9.514271	4.838393
H	0.784174	-8.461960	3.870532
H	0.448251	-8.469867	6.895241
H	1.880405	-8.049645	5.936788
H	-2.025881	-7.824677	4.855061
H	-1.192987	-7.214096	3.427939
H	1.737323	-6.225736	7.552536
H	1.542610	-4.679946	4.351465
H	2.288813	-3.981666	7.230005
H	5.713013	-7.578022	5.748762
H	5.921809	-6.336205	4.515138
H	4.001481	-6.301909	6.870554
H	4.862897	-4.948512	6.117401
H	3.754414	-8.483478	4.594674
H	4.577084	-7.828666	3.187899
H	0.206050	-5.384829	7.764315
H	0.814257	-3.589152	6.353944
H	1.337189	-1.903129	4.598751
H	3.938716	-3.450697	4.140114
H	1.716258	-4.818222	0.217237
H	1.477236	-3.090827	-0.078821
H	2.875633	-4.434408	2.262593
H	3.316532	-2.908461	1.454100
H	-0.117783	-4.878474	1.825573
H	-0.724277	-3.556867	0.818042
H	3.607787	-0.664456	4.585747
H	3.735413	-1.065485	2.890922
H	3.449534	-2.703853	5.652692
H	3.910972	1.536369	2.766402
H	1.164888	-0.054073	-1.116182
H	1.127142	1.569844	-0.427676
H	2.480829	-0.902491	0.725307
H	3.019786	0.788399	0.783436
H	-0.612871	-0.805129	0.392563
H	-1.067321	0.900616	0.309907
H	1.395842	0.458753	4.168648
H	3.455852	1.761093	4.455809
H	2.479115	2.506124	3.180377

PVP-Ca-TiO_2-II

C	-0.011494	0.003038	0.004088
N	-0.024179	-0.006681	1.480918
C	1.203122	-0.024416	1.990356
C	2.217829	-0.074559	0.886817
C	1.396067	-0.487093	-0.332087
C	-1.271717	0.045118	2.264458
C	-2.075146	-1.226531	2.024158
O	1.478276	0.056748	3.216133
C	-2.096872	1.288062	1.915775
C	-1.816200	2.606311	2.644836
N	-2.374579	2.588830	4.017624
C	-1.843263	2.127015	5.147381
C	-2.841327	2.229597	6.264893
C	-3.903772	3.175632	5.710269
C	-3.788711	2.985137	4.202137
O	-0.679228	1.666634	5.272109
C	-0.376938	3.116797	2.532043
C	-0.010298	4.289620	3.449331
N	1.382248	4.693815	3.194172
C	2.428582	4.114862	3.785477
C	3.696675	4.793816	3.363722
C	3.282776	5.560482	2.107204
C	1.776024	5.775317	2.273384
O	2.342965	3.150077	4.584926
C	-0.958327	5.471254	3.342542
C	-0.896285	6.367866	4.573275
N	-1.630985	7.622211	4.452741
C	-1.000608	8.918535	4.746699
C	-2.179691	9.891779	4.755270
C	-3.219109	9.211430	3.863784
C	-2.836173	7.767365	3.909450
O	-3.537187	6.806690	3.485932
Ti	-3.868141	6.437657	1.497740
O	-5.399571	5.331335	1.924109
Ti	-5.770366	4.172635	0.657315
O	-7.422226	4.303991	-0.167235
Ti	-6.936228	5.244205	-1.647836
O	-6.750933	7.025990	-1.605165
Ti	-4.964821	7.554598	-1.498375
O	-4.426911	9.200234	-2.085346
Ti	-2.625761	9.077854	-2.409336
O	-1.309492	9.408032	-1.168373
Ti	-1.389486	7.803229	-0.227180
O	-0.362620	6.225855	-0.400897

Ti	-0.884635	4.924086	-1.538021
O	-0.726360	5.662614	-3.158803
Ti	-2.488154	6.308284	-3.446080
O	-3.258194	4.950761	-4.361194
Ti	-4.531677	4.532174	-3.068911
O	-4.453693	6.483062	-2.944296
O	-4.702968	7.490302	0.319265
O	-2.171859	7.781950	1.289999
O	-3.001672	5.123261	0.787862
Ti	-3.223020	3.611091	-0.644175
O	-1.415505	3.272670	-0.704842
O	-2.720536	7.450750	-1.493560
O	-2.277231	8.124627	-3.888991
O	-2.672210	5.116817	-1.789004
O	-3.973875	3.021449	-2.200359
O	-5.164674	5.009098	-0.945324
O	-4.482423	2.888157	0.564564
O	-6.361901	4.478186	-3.189412
Ca	1.484331	1.156502	5.150079
H	-1.895636	10.886526	4.405690
H	-2.565204	9.997164	5.775141
H	-0.273711	9.141041	3.949502
H	-0.465549	8.871200	5.701354
H	-3.153144	9.519066	2.810157
H	-4.257816	9.346935	4.179774
H	-1.273908	5.816082	5.450499
H	-0.017160	3.932173	4.491165
H	-0.790814	6.041633	2.418809
H	3.822618	6.500785	1.976889
H	3.473329	4.949893	1.217295
H	1.539881	6.749519	2.729195
H	1.213299	5.704687	1.333948
H	4.012882	5.456558	4.181952
H	4.503714	4.070734	3.215674
H	0.143028	6.640934	4.803891
H	-1.971443	5.077782	3.250403
H	-2.445423	3.352029	2.139068
H	0.350397	2.312337	2.699922
H	-4.909222	2.973780	6.085400
H	-3.657426	4.214332	5.965441
H	-4.437994	2.182726	3.822240
H	-4.005741	3.891677	3.622882
H	-3.236709	1.220443	6.453173
H	-2.362068	2.558698	7.192018
H	-3.159358	1.055235	2.064260
H	-2.003921	1.480848	0.838705

H	-0.263259	3.423814	1.478678
H	-2.504255	-1.248643	1.015294
H	1.767094	-0.070578	-1.270917
H	1.398034	-1.578200	-0.430671
H	-0.204536	1.026733	-0.351943
H	-0.800483	-0.646547	-0.387438
H	2.642585	0.937047	0.785580
H	3.044848	-0.744495	1.139155
H	-0.957907	0.073229	3.315669
H	-2.911349	-1.277759	2.729256
H	-1.457694	-2.120487	2.159724
